# Employment Status and HIV Viral Load in Chilean Adult Population: A Propensity Score Analysis

**DOI:** 10.1007/s10461-024-04600-y

**Published:** 2025-01-09

**Authors:** Ignacio Leiva-Escobar, Claudia P. Cortes, Angelo Lamadrid

**Affiliations:** 1 Internal Medicine IX-Department of Clinical Pharmacology and Pharmacoepidemiology, Medical Faculty Heidelberg/Heidelberg University Hospital, Heidelberg University, Heidelberg, Germany; 2 Medicine Department, School of Medicine, Universidad de Chile, Santiago, Chile; 3 Fundación Arriarán, Hospital Clínico San Borja-Arriarán, Santiago, Chile; 4 Centre for HIV/AIDS Integral Research -CHAIR, Faculty of Medicine, Universidad de Chile, Santiago, Chile; 5 Department of Health Care Management, Technische Universität Berlin, Berlin, Germany

**Keywords:** HIV Treatment Adherence, Social Determinants of Health, Chile, Latin America, Unemployment

## Abstract

We set out to investigate the potential impact of unemployment on HIV viral load in individuals living with HIV at the biggest HIV-related healthcare centre in Chile. We analysed a cross-sectional dataset of 803 adults living with HIV on antiretroviral therapy. The main exposure was employment status. The outcome, detectable HIV viral load, was operationalised using a cut-off of HIV viral load at 20 copies/mL. We applied a propensity score method, the inverse probability of treatment weighting to control for measured confounders. We found that 219 (27.3%) of participants were unemployed. Being unemployed was associated with increased odds of being detectable (OR = 1.78, 95%CI = 1.18–2.71) compared to being employed. Additionally, we found that those unemployed and non-adherents have higher odds of being detectable (OR = 2.53, 95%CI = 1.18–5.41). Unemployment status may influence HIV viral load. However, further research is needed to determine and understand the social structure behind those relationships in the Chilean people living with HIV.

## Resumen

Nos propusimos investigar el potencial impacto del desempleo en la supresión viral de VIH en personas viviendo con VIH en uno de los centros más grandes de Chile. Analizamos una base de datos transversal de 803 adultos en tratamiento antirretroviral donde el principal factor de exposición fue el estado de empleo del participante y el desenlace, la detectabilidad de la carga viral de VIH (≥ 20 copias/mL). Aplicamos la ponderación por el inverso de la probabilidad de tratamiento para controlar por los confusores. De los participantes, 219 (27.3%) estaban desempleados. El desempleo se asoció con un aumento en los odds de estar detectable, en comparación con estar empleado (OR = 1.78, 95%CI = 1.18–2.71). Adicionalmente, encontramos que aquellos participantes desempleados y no adherentes tuvieron mayores odds de estar detectables (OR = 2.53, 95%CI = 1.18–5.41). El desempleo puede influenciar la supresión de la carga viral de VIH. Sin embargo, se necesita más estudios para determinar y comprender la estructura social de esta relación en la población chilena viviendo con VIH.

## Introduction

Ending the AIDS epidemic by 2030 is one of the sustainable development goals. To achieve this, the Joint United Nations Programme on HIV/AIDS (UNAIDS) proposed intermediate goals through the strategy “90-90-90” and then progressively increased the approach to “95-95-95”, meaning that 95% of people living with HIV should know their status, 95% of them should be on antiretroviral therapy (ART), and 95% of people on ART achieve a suppressed or undetectable HIV viral load [1].

Treatment with ART and reaching an undetectable HIV-viral load are crucial pillars to controlling the epidemic by 2030. Timely access to ART has shown effectiveness in improving health outcomes and reducing HIV transmission [2, 3]. In fact, people who achieve and maintain a virally suppressed status prevent disease progression and do not sexually transmit HIV to others, a phenomenon known by the phrase *undetectable = untransmittable* [4–7].

Different factors, such as the combination of antiretroviral drugs and the patient’s clinical features, might affect HIV viral load [8]. However, adherence plays an important role, as adequate and constant antiretroviral plasma levels are needed to suppress virus replication and prevent drug resistance [9–11]. Studies have reported that adherence to ART $\:\ge\:$ 95% is required to achieve and maintain HIV viral suppression [12, 13].

The World Health Organization (WHO) defined the Social Determinants of Health as *“the conditions in which people are born*,* grow*,* work*,* live and age*,* and the wider set of forces and systems shaping the conditions of daily life”* [14]. HIV viral suppression is associated with social determinants of health because these define health behaviours such as adherence to ART, resulting in negative or positive health outcomes (HIV non-viral suppression v/s viral suppression) [15].

Employment is considered a social determinant of health because employed people could have higher economic security, social networks, and well-being [14]. The lack of these benefits could expose unemployed people to circumstances that would have detrimental impacts on their health status. This association might vary depending on the context, individual, and health outcome. Researchers have agreed that the mechanism behind measuring employment as a social determinant of health is very complex to analyse [16, 17].

The specific association between HIV viral load and employment is unclear. While a recent systematic review and meta-analysis states that it is unable to establish an association between them in high-income countries [18], other studies claim that employed people are 27% more likely to adhere to HIV treatment than unemployed [19] and therefore being virally suppressed.

Observational studies play a crucial role in generating evidence to inform the decision-making process in public health, especially when conducting randomised controlled trials is impractical or unethical. However, observational studies are prone to confounding and selection bias, requiring more sophisticated statistical methods to address these challenges. Propensity score (PS) methods have been used to improve the validity of causal inferences from observational data by mimicking the randomisation process, making participants more comparable regarding observed characteristics [20, 21].

### Chile and HIV

Chile is considered one of the few high-income countries in Latin America by the World Bank, but with deep socio-economic inequalities [22] and health outcomes similar to middle-income countries [23].

Regarding HIV, the country has not followed the global downward trend of new HIV infections. Meanwhile globally, new infections decreased by 38% between 2010 and 2022, in Chile, it increased by 59% in the same period [24].

In 2022, UNAIDS estimated 4800 new infections in Chile. Additionally, 94% of the people living with HIV knew their status (78170), 74% of the people living with HIV were on treatment (61502), and 72% of the people living with HIV were virally suppressed (59564) [24].

The highest prevalence is reported among men who have sex with men (MSM). Meanwhile, in the general population aged between 15 and 49, the prevalence is 0.5%; among homosexual, bisexual and other MSM, it rises to 17.6% [25]. On the other hand, the transmission rate for the use of injected drugs is close to zero [26].

Chile has public and private health insurance. Both are under the GES regimen (Garantias Explicitas en Salud/Explicit Health Guarantees) that ensures timely, affordable, and quality care for 87 prioritised diseases, including HIV [23, 27]. Over 78% of the population is enrolled in public health insurance [23], which provides all HIV services free of charge to their beneficiaries [27].

Considering the Chilean scenario and that few of these studies have been conducted in the Latin American region [11], we aimed to determine whether employment status affects HIV viral load in a high-income Latin American country (Chile) using a PS analysis. Our findings will support the Chilean government and others in reaching the sustainable development goal in order to end the HIV epidemic by 2030 and develop a more integral approach, including social determinants of health in the management of HIV care.

## Methods

### Study Population

This cross-sectional analysis was carried out at the Fundación Arriarán in Santiago (capital city), which is the largest HIV healthcare centre in the country. It provides medical consultations, laboratory test sampling, and ART to 5160 people living with HIV enrolled in public health insurance.

### Data Sources

#### Survey Data

Outpatients from Fundación Arriarán were asked to reply the “Computer platform based on artificial intelligence for characterisation and identification of the degree of adherence of the population with HIV” self-administered online survey, which was made up of 7 items (employment and housing status, HIV treatment, consumption of alcohol and drugs, HIV stigma, mood and social relations, daily life, and additional information). In this study, we used the items of employment and housing status and consumption of alcohol and drugs. 827 participants agreed to participate and responded to the survey.

Between February to June 2021, data was collected in Spanish language. Participants accessed the survey through a QR code published inside the health centre facility, and those without access to the QR code were provided with a paper-based survey. Participants identified themselves, indicating the initials of their name and date of birth.

#### Pharmacy and Clinical Records

We use pharmacy and clinical records to complement the participant information. Records were matched based on the initials of the participant’s name and date of birth. After this matching process, we pseudonymised participants and excluded those not identified from this study.

### Inclusion and Exclusion Criteria

The inclusion criteria of the sample were: (a) patients $\:\ge\:$ 18 years, (b) patients who started ART at least six months before responding to the survey and had at least one HIV viral load measurement after initiating therapy, and (c) patients who responded to questions regarding the employment status.

Exclusion criteria were: (a) patients who started ART being pregnant. Since they are follow-up according to different protocols, (b) patients who re-started therapy or came from another health centre during the period studied, and (c) patients enrolled in a clinical trial.

### Outcome

Our outcome was being HIV virally detectable (hereafter detectable), which was defined as HIV viral load ≥ 20 copies/mL, the cut-off used in Chile based on the HIV RNA Assay Roche COBAS 6800 [28]. The data was obtained from clinical records. All individuals starting ART are tested on average three months later, then every six months. We retrieved the last test in a six-month period before the index date (i.e., the survey date).

### Exposure and Covariates

Table 1 lists and describes the operationalisation of exposure and covariates. Baseline covariates were retrieved before the index date.

**Table 1:** Definition and operationalisation of variables

Variable	Definition	Data Source	Categories
**Outcome**			
HIV viral load (VL) (being HIV virally detectable)	HIV viral load in blood (cut-off of 20 copies/mL at the last test in a six-month period before the survey).	Clinical records	1. Being detectable: patients with VL ≥ 20 copies/ mL 2. Being undetectable: patients with VL < 20 copies/ mL
**Exposure**			
Employment status	Employment status at the time of the survey	Survey data	1. Employed 2. Unemployed (people unemployed, retired or students)
**Covariates**			
Gender	Gender self-reported	Pharmacy records	1. Women 2. Men
Age	Derived from the date of birth and the index date	Pharmacy records	It was considered as a continuous variable.
Health insurance classification	Based on the Public National Health Found (Fondo Nacional de Salud) stratification	Pharmacy records	1. A/ B: People with the lowest income 2. C/ D: people with higher income.
Additional long-term conditions	Any additional long-term conditions the patient had.	Survey data, Clinical records	1. No: No additional chronic diseases 2. Yes: ≥ 1 additional chronic disease
Migrant background	Documented information	Pharmacy records	1. Chilean 2. Another nationality
Educational level	Education level self-declared.	Pharmacy records	1. Primary 2. Secondary 3. Tertiary
Time on antiretroviral treatment	Time since initiation of antiretroviral therapy for a treatment-naive patient to the index date., in years	Pharmacy and Clinical records	1. Patients on treatment for ≤ 1 year 2. Patients on treatment for > 1 year
Type of antiretroviral treatment	Based on the last dispensation prior to the index date	Pharmacy records	1. Single-tablet regimen (STR) 2. Multiple-tablet regimen (MTR)
Municipality of residence	If the participant resides in the same municipality as the health centre	Pharmacy records	1. Santiago 2. Others
Housing status	Self-reported house status	Survey data	1. Living in owned or rented housing 2. Shelter, freeloader, homeless
Self-stigma	Do you agree with the following sentences: • I am very careful about whom I tell about my HIV status. • I am not as good as others because of my HIV status. • Many people with HIV are rejected when others become aware of their disease.	Survey data	1. Disagree or fully disagree with the three questions 2. Agree or fully agree on at least one of the questions.
Addiction	• In the last year, how often did you use any kind of drug (e.g. marijuana, poppers, cocaine, ecstasy, ketamine, G, etc.) or alcohol to change your mood? • In the last year, how often did you use more drugs or drink more alcohol than you planned? • In the last year, how often did you feel the need or want to stop using drugs or alcohol, and you could not?	Survey data	1. People who answered “never” in the three questions 2. People who answered “at least one per week”, “at least one per day”, “more than one time per month”, or “lower than one time per month” in at least one of the questions.
Mental health	• How often did you feel less interest or pleasure in your daily activities in the last two weeks? • In the last two weeks, how often did you feel depressed, annoyed, or hopeless?	Survey data	1. People who answered “no day” in the two questions 2. People who answered “several days”, “more than half of the days”, or “almost every day” in at least one of the questions.
Proportion of days covered (PDC) (being non-adherent)	A proxy for adherence was calculated based on the number of days the patient received medication six months before the survey—a cut-off of 95%.	Pharmacy records	1. Adherence: Patients with a percentage ≥ 95% 2. Non-Adherence: Patients with a percentage < 95%.

### Statistical Analysis

We used propensity score (PS) to obtain the inverse probability of treatment weighting (IPTW) to create a pseudo population in which both groups (unemployed and employed) were balanced in terms of baseline characteristics. PS was defined as the probability of being unemployed given all the baseline covariates, including age, gender, health insurance classification, additional long-term conditions, migrant background, educational level, time on ART, type of ART, municipality of residence, housing status, proportion of days covered (PDC), self-stigma, addictions, and mental health. We applied a gradient boosted model, a multivariate nonparametric method that involves an iterative process with multiple regression trees (10000) to capture complex and non-linear relationships between employment status and the baseline covariates [29, 30]. We calculated the stabilised weights by taking the probability of being unemployed without considering covariates (i.e., marginal probability) in the numerator to avoid extreme values [31]. Additionally, IPTW enables to retain most individuals in the analysis which also yields stable standard errors [32].

We assessed balance covariate between the unemployed and employed groups using absolute standardised mean differences (ASMD) where a value less or equal to 0.1 was considered a good balance [33].

Our analysis estimated the marginal odds ratios for being detectable using logistic regression. We included only employment status as a covariate and applied stabilised weights to the model. We utilised bootstrapping with 5000 iterations to estimate the confidence intervals, as the weights reduce the precision of standard errors [34]. Furthermore, we conducted an interaction analysis by examining the interaction between employment status and adherence.

All analyses were conducted in the R version 4.2.2, and the Toolkit for Weighting and Analysis of Non-equivalent Groups (TWANG) package was used to estimate the PS [35].

### Consent

All patients gave their written consent to participate.

## Results

### Descriptive Statistics

From a total of 827 participants who responded to the survey, 20 were not identified, 3 were duplicates, and one did not have pharmacy records. After applying inclusion/exclusion criteria, we did not find any missing data.

A summary of participants’ baseline characteristics stratified by employment status, both before and after weighting, is shown in Table 2. Of the participants, 584 (72.7%) were employed. The mean participant age in the unweighted cohort was 40.5 (SD = 12.5) years among the unemployed and 38.6 (SD = 9.5) among the employed. The largest number of participants were men, 195 (89.0%) in the unemployed group and 561 (96.1%) in the employed group. Employed individuals also belonged to the highest health insurance classification, had a greater level of education, and owned or rented their housing.

**Table 2:** Characteristics of the participants (*n* = 803)

Characteristic	Unweighted cohort			Weighted cohort		
	Employed *N* = 584 (72.7%)	Unemployed *N* = 219 (27.3%)	ASMD	Employed *N* = 542.8 (76.1%)	Unemployed *N* = 170.9 (23.9%)	ASMD
Men – no. (%)	561.0 (96.1)	195.0 (89.0)	0.270	517.1 (95.3)	159.5 (93.3)	0.084
Age in years – mean (SD)	38.6 (9.5)	40.5 (12.5)	0.173	38.8 (9.9)	39.0 (10.7)	0.013
Health insurance classification – no. (%)			0.253			0.033
A and B	268.0 (45.9)	128.0 (58.4)		259.4 (47.8)	84.5 (49.4)	
C and D	316.0 (54.1)	91.0 (41.6)		283.3 (52.2)	86.4 (50.6)	
Additional long-term conditions – no. (%)	173.0 (29.6)	72.0 (32.9)	0.070	161.6 (29.8)	50.9 (29.8)	0.000
Migrant background – no. (%)	165.0 (28.3)	50.0 (22.8)	0.125	148.6 (27.4)	46.7 (27.3)	0.001
Education level – no. (%)						
Primary	10.0 (1.7)	10.0 (4.6)	0.301	10.6 (2.0)	4.4 (2.6)	0.055
Secondary	152.0 (26.0)	80.0 (36.5)		151.8 (28.0)	50.2 (29.4)	
Tertiary	422.0 (72.3)	129.0 (58.9)		380.3 (70.1)	116.3 (68.0)	
≥ 1-year on antiretroviral treatment – no. (%)	556.0 (95.2)	207.0 (94.5)	0.031	517.0 (95.2)	163.8 (95.8)	0.028
Living in the same municipality as the health centre – no. (%)	325.0 (55.7)	116.0 (53.0)	0.054	299.3 (55.1)	97.2 (56.9)	0.035
Living in owned or rented housing – no. (%)	462.0 (79.1)	125.0 (57.1)	0.486	408.7 (75.3)	121.9 (71.3)	0.090
At least one question related to self-stigma – no. (%)	575.0 (98.5)	212.0 (96.8)	0.109	532.8 (98.2)	167.5 (98.0)	0.011
At least one question related to addiction (alcohol/drugs) – no. (%)	392.0 (67.1)	131.0 (59.8)	0.152	358.6 (66.1)	111.0 (64.9)	0.024
At least one question related to mental health – no. (%)	368.0 (63.0)	151.0 (68.9)	0.126	347.4 (64.0)	109.3 (63.9)	0.001
Proportion of days covered (PDC) ≥ 95% – no. (%)	414.0 (70.9)	163.0 (74.4)	0.040	396.5 (73.1)	132.2 (77.4)	0.067
Single tablet regimen – no. (%)	360.0 (61.6)	130.0 (59.4)	0.047	330.6 (60.9)	105.5 (61.7)	0.017

### Comparison before and after Covariate Balancing

**Fig. 1:**
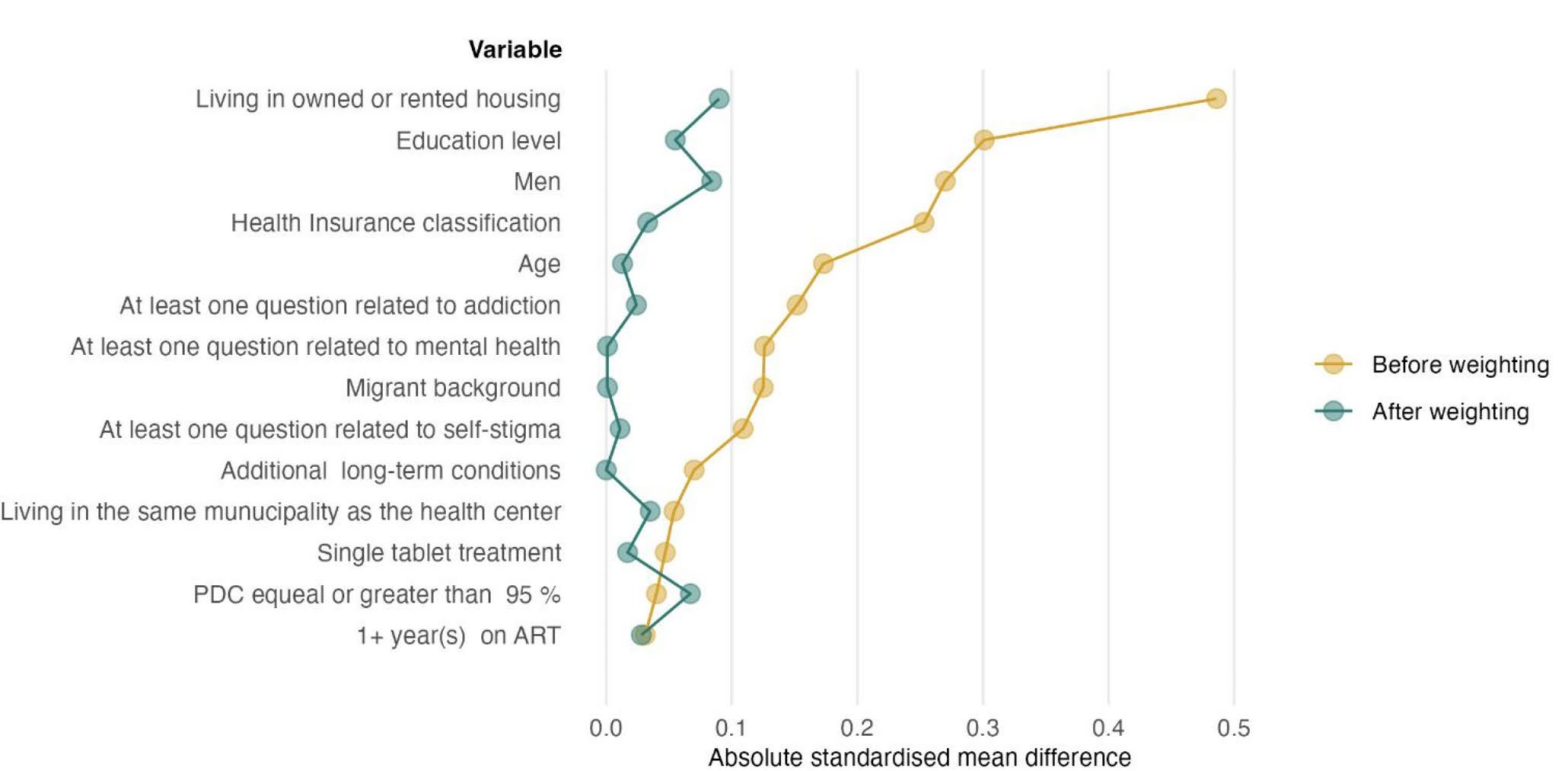
Loveplot showing the absolute standardise mean differences of covariates before (yellow) and after (green) applying propensity score

**Fig. 2:**
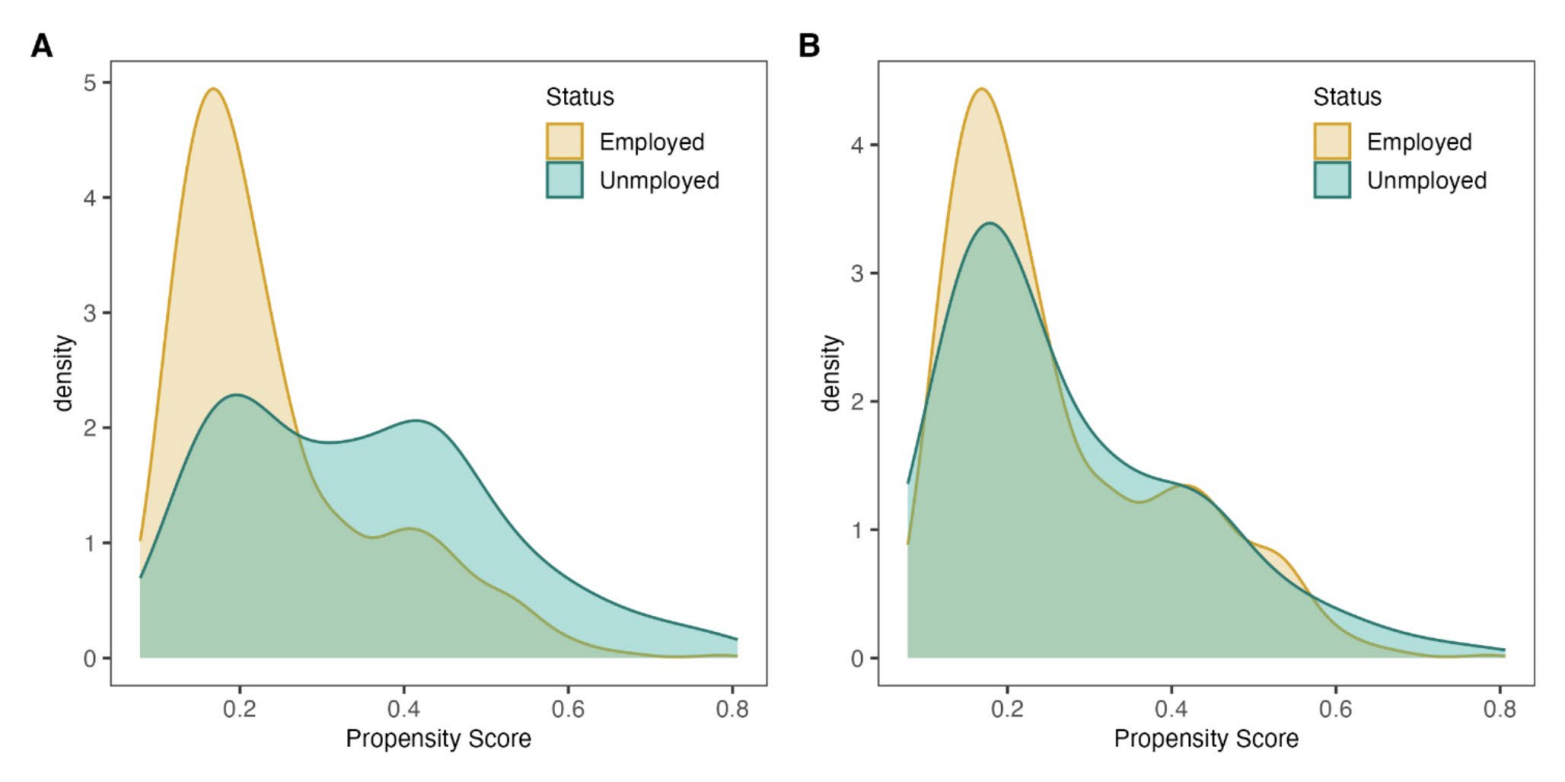
**Panel A**: Density plot illustrating the distribution of propensity scores for unemployed and employed individuals, highlighting their overlap. **Panel B**: Density plot displaying the distribution of propensity scores for unemployed and employed individuals, adjusted for weights to reflect weighted densities

We found an imbalance in 9 out of 14 covariates with ASMD > 0.1 between the employed and unemployed groups (Table 2). Among those, gender, health insurance classification, educational level, and living in owned or rented housing presented the greatest differences. After weighting the cohort, all covariates’ differences decreased below 0.1, showing an adequate balance (Table 2; Fig. 1). Figure 2 visually presents the necessary overlap in propensity scores to ensure comparability between employed and unemployed participants. The overlap improves significantly with the weighted densities, indicating that the groups are becoming more comparable.

### Unemployment Effect

Of the unemployed participants, 56 (25.6%) were detectable (i.e., HIV viral load ≥ 20 copies/mL), while of the employed participants, 84 (14.4%) were detectable.

Table 3 shows the unadjusted and adjusted odds ratio (uOR and aOR, respectively) for being detectable. The uOR was 2.04 (95%CI = 1.40–3.00), which decreased to 1.78 (95%CI = 1.18–2.71) after adjustment. This indicates that being unemployed was associated with a statistically significant 78% increase in the odds of being detectable.

Considering participants’ adherence, the OR for being detectable among unemployed adherents compared to employed adherents was 1.87 (95%CI = 1.07–3.27). In contrast, the OR for being detectable among unemployed non-adherent compared to employed non-adherent was 2.53 (95%CI = 1.18–5.41). This suggests that unemployed non-adherents have 35.29% higher odds of being detectable compared to unemployed adherent participants.

**Table 3:** (A) Unadjusted and adjusted (IPTW) odds ratios of being detectable. (B) Odds ratios of being detectable within individuals with ≥ 95% and < 95% ART adherence based on the proportion of days covered (PDC)

Model	Odds Ratios (95%CI)
A)	
Unadjusted, unemployed vs. employed	2.04 (1.40–3.00)
Adjusted, unemployed vs. employed	1.78 (1.18 – 2.71)
B)	
Unemployed vs. employed, among the adherent	1.87(1.07–3.27)
Unemployed vs. employed among the non-adherent	2.53 (1.18–5.41)

## Discussion

We set out to evaluate the effect of employment status on HIV viral load in a cohort of adults living with HIV. We found that being unemployed has a negative effect on participants’ HIV viral load. Unemployed participants were more likely to be detectable (aOR = 1.78, 95%CI = 1.18–2.71) compared to employed participants.

Additionally, non-adherence negatively affected the association between employment status and HIV viral load since the OR of being detectable among unemployed non-adherent participants increased by 42.13% when compared to only being unemployed.

This is likely because ART adherence mediates the relationship between the exposures studied and HIV viral load; most factors that may influence adherence can also affect detectability [36].

Our findings align with a few previous studies that investigated the effect of employment on viral suppression in other high-income countries [37, 38]. For instance, a study conducted in the United States claims that people with full-time work have 4.03 higher odds (95%CI = 1.27–12.83) of being virally suppressed compared to people who are unemployed [38].

In the general population, employment also has a protective effect, especially on depression and mental health [39]. This could be explained by Jahoda’s latent deprivation model [40, 41], which proposes that employment provides manifest and latent functions. The former is related to people’s income, and the latter corresponds with psychological needs [42]. Thus, unemployed people who lack these benefits might be exposed to unfavourable circumstances at financial and psychological levels [16, 17].

According to Jahoda, on the one hand, unemployment might affect health through socio-economic status, poverty, and financial anxiety [17, 43]. In turn, poverty has been described by Marmot [44] as a social determinant of health associated with material deprivation and limited social participation, resulting in a narrow capacity to take control over one’s life. For all these reasons, unemployment is considered one of the most important socio-economic determinants of quality of life among people living with HIV [45], and it could play a key role in health behaviours related to HIV viral suppression and undetectability [37, 38].

However, unemployment as a risk factor for being detectable in the Chilean population is not fully supported, at least by the manifest functions of employment according to the latent deprivation model, because in the country, HIV diagnosis, medical care and antiretroviral treatment are free of charge to people belonging to the national public health insurance [46].

Another study supporting this idea was based on the United Kingdom [47], a country with universal free access to health care (including HIV services), displayed that among unemployed people, the prevalence of being non-virally suppressed is higher compared to employed ones, which points out that the association between unemployment and higher HIV viral load is far from being exclusively explained by lack of income to access HIV medical care or treatment [36].

On the other hand, employment could satisfy psychological needs and, in this way, maintain a status of higher well-being [42]. Therefore, the social and psychological distress caused by unemployment could address detrimental health behaviours, such as poor self-care, substance abuse [48–50], and reduction of treatment adherence [51]. In fact, people living with HIV who lose their jobs have been associated with episodes and/or long-term periods of HIV viral load higher than 10,000 copies/mL [15].

Previous research states that mental health in people living with HIV is already engaged. For instance, Feullet [52] claims that men and women with HIV have 1.55 to 5.12 and 1.94-to-2.61-fold higher prevalence of major depressive symptoms, respectively, compared with the general population of the same gender, which has been associated with experiences of discrimination, stigma and disclosure of HIV status [52].

Unemployment (as well as non-paid employment) increases the level of stress and depressive symptoms due to loss of income and total or partial lack of participation in social activities [53], which would worsen mental health status among people living with HIV and consequently, the adherence to HIV treatment and HIV viral load. In fact, a study assessing adherence to antiretroviral therapy and depression in Chile showed that participants with moderate/severe depression have 3.08 (95%CI = 1.08–8.80) higher odds of being non-adherent compared with participants with mild to non-depression [54].

Additionally, according to the WHO, patient-related factors, such as life stress, depression, hopelessness, and use of alcohol and drugs, are considered one of the most important drivers associated with adherence to ART and, consequently, HIV viral load [55].

It is noteworthy to clarify that employment is not always a protective factor for mental health. People exposed to insecure working conditions, high psychological demands, and little decision-making authority tend to report similar levels of low well-being and high depressive symptoms as the unemployed [15, 56]. Additionally, attitudes towards stigma and discrimination against people living with HIV for other workers also have a negative impact on mental health in this group [57].

To our knowledge, this is the first study conducted in Latin America assessing the effect of employment status on HIV viral load applying PS methods to minimise confounding effectively. However, we acknowledge limitations which need to be considered. The survey was entirely voluntary, which could introduce selection bias, as it may underrepresent participants with poor health performance. Moreover, healthcare providers’ expectations could influence participants’ responses, introducing desirability bias.

Additionally, our analysis was conducted on cross-sectional data, making it challenging to establish a causal effect of employment status on HIV viral load due to the nature of the data. We only adjusted for measured confounding, and the presence of unmeasured confounders is highly likely.

Despite our cohort being balanced across various social, addiction, and psychological characteristics, it is possible that some of them may not be comprehensively captured or represented accurately. Consequently, additional information must be considered to effectively determine the underlying effect of employment status on HIV viral load.

We used an indirect-objective method to assess adherence (proportion of days covered), which cannot inform whether patients actually took their medication as prescribed, introducing misclassification if adherence was not correctly captured.

When comparing our results with those of other studies, it is worth noting that we consider a low cut-off point for detectability based on the techniques applied in the country and in line with the updated definition of undetectability published by the WHO [58].

Finally, it is important to underline that this study was conducted during the COVID-19 pandemic, which may have affected multiple aspects of people’s lives. Therefore, readers should consider these limitations when interpreting our findings.

## Conclusion

Our findings suggest that employment status impacts HIV viral load among people on ART. The association between employment status and undetectability may be affected by the level of adherence to ART, where unemployed individuals showed higher odds of being detectable when having a lower level of adherence.

These findings underline the importance of exploring the complex social structure underpinning the studied relationship, providing a compelling ground for further studies. Additionally, policymakers and clinicians should consider social determinants in the management of HIV as they are essential components to deal with the disease, advocate for policy changes and move forward with the elimination of HIV by 2030.

## Data Availability

Not publicly available.
